# Enriching Hippocampal Memory Function in Older Adults Through Real-World Exploration

**DOI:** 10.3389/fnagi.2020.00158

**Published:** 2020-06-03

**Authors:** Branden S. Kolarik, Shauna M. Stark, Craig E. L. Stark

**Affiliations:** Department of Neurobiology and Behavior, University of California, Irvine, Irvine, CA, United States

**Keywords:** environmental enrichment, aging, spatial exploration, pattern separation, intervention

## Abstract

Age-related structural and functional changes in the hippocampus can have a severe impact on hippocampal-dependent memory performance. Here, we tested the hypothesis that a real-world spatial exploration and learning intervention would improve hippocampal-dependent memory performance in healthy older adults. We developed a scavenger hunt task that participants performed over the course of a 4-week behavioral intervention period. Following this intervention, participants’ lure discrimination index (LDI) on the Mnemonic Similarity Task was significantly higher than it was at baseline and greater than that of a No-Contact Control Group, while traditional recognition scores remained relatively unchanged. These results point to the viability of a spatial exploration intervention for improving hippocampal-dependent memory in older adults.

## Introduction

Environmental enrichment provides animals with an environment that allows for physical exercise and exploration. Exposure to an enriched environment can promote neurogenesis, synaptogenesis and improve performance on hippocampal-dependent memory tasks (Kempermann et al., 1997; van Praag et al., 1999; Clemenson et al., 2015). One important component of environmental enrichment appears to be the amount spatial exploration of the environment. Animals that spend more time exploring their environment show increased neurogenesis in the dentate gyrus of the hippocampus (Freund et al., 2013). In humans, extensive spatial exploration training has been linked to increased hippocampal volume in real (Maguire et al., 2000) and virtual environments (West et al., 2017) as well as the slowing of age-related decline (Lövdén et al., 2012). Normal healthy aging can also bring about structural and functional changes in the hippocampus (Raz et al., 2005; O’Shea et al., 2016) and can have a profound impact on hippocampal-dependent memory performance (Yassa et al., 2011; Konishi et al., 2017). In animals, however, these changes can be partially alleviated through environmental enrichment (Kempermann et al., 1998; Fernandez et al., 2004; Bennett et al., 2006). Given our understanding of age-related structural and behavioral changes, as well as the impact of environmental enrichment on the hippocampus, how can we promote memory improvement in humans with environmental enrichment?

We have previously explored the use of 3D video games as a means of providing environmental enrichment. Video games, particularly 3D video games that allow for spatial exploration of a virtual environment, are a way for humans to interact with a novel, enriched environment and learn the spatial layout of landmarks and goal locations. Previous work from our lab, using video games as a behavioral intervention, showed that playing games for 30 min per day for 2 weeks improves hippocampal-based mnemonic pattern separation in young adults (Clemenson and Stark, 2015; Clemenson et al., 2019). Importantly, this effect was present specifically for those who played a rich 3D rather than a simple 2D video game (Clemenson and Stark, 2015) and correlated with their amount of spatial exploration (Clemenson et al., 2019). Recently, we have found similar improvements in hippocampal-based memory performance in older adults who played a rich 3D game in a 4-week intervention (Clemenson et al., 2020). These results suggest that the spatial exploration of a virtual environment can promote changes in hippocampal-dependent memory tasks, similar to those seen previously in rodents.

In the current study, we set out to investigate if a real-world spatial exploration task, similar to that used in the video game interventions, can also have an impact on hippocampal-based memory function in healthy older adults. We developed a scavenger hunt task that had participants learning arbitrary locations within local parks over the course of a 4-week behavioral intervention period. We assessed hippocampal memory function with the Mnemonic Similarity Task (MST) (Kirwan and Stark, 2007; Stark et al., 2013, 2019) before and after our intervention as well as at a 4-week follow-up test. We focused on the MST’s lure discrimination index (LDI) as our primary endpoint. The MST’s LDI tests object recognition memory using highly similar lure items in an effort to tax the hippocampal function known as pattern separation (Yassa and Stark, 2011; Rolls, 2016). The MST LDI has been shown to be highly sensitive to hippocampal function (Kirwan and Stark, 2007; Stark et al., 2013), to age-related memory decline (Toner et al., 2009; Holden et al., 2013; Stark et al., 2013, 2015; Stark and Stark, 2017) and to hippocampal connectivity (Yassa et al., 2010, 2011; Bennett and Stark, 2015; Bennett et al., 2015) and function (see Stark et al., 2019 for review). Since environmental enrichment and spatial exploration have been shown to specifically affect the hippocampus in rodent models, we hypothesized that our real-world exploration intervention should have the most effect on hippocampal-dependent memory (MST LDI) and little effect on traditional recognition memory (MST REC). These two metrics provide a valuable contrast because simple object recognition memory is not heavily impacted by hippocampal function, while LDI performance is critically dependent on hippocampal integrity. For example, patients with hippocampal damage are seemingly unimpaired relative to matched controls for REC while displaying strong impairments in LDI (Kirwan et al., 2012).

Here, we investigated whether mnemonic discrimination (MST LDI) significantly improved following a real-world exploration intervention, both immediately following the intervention and then following a 4 week washout period. We further evaluated whether any change in memory ability was related to pre-test baseline scores, the rate of learning in the behavioral intervention, or the amount of physical activity completed during the intervention. While we have previously not observed test-retest effects of the MST (Stark et al., 2015), we also included a No-Contact Control Group that completed the MST with a 30-day intervening delay for comparison.

## Materials and Methods

### Participants

Fifty-six adults between the ages of 65-89 were recruited from the community around UC Irvine and screened for history of neurological disease or psychiatric illness, spoke fluent English, had normal or corrected-to-normal vision, and were able to sustain light activity (e.g., walking) for 30-min periods. Eight participants dropped out before completing the study, so our final sample included 25 participants (mean age = 69.8 years; range = 61–76; standard deviation = 4.1; 20 Female) in the Intervention Group and 23 participants (mean age = 71.4; range = 60–89; standard deviation = 8.4; 18 Female) in the No-Contact Control Group. To determine cognitive status, all participants also completed the Mini Mental State Exam (Folstein et al., 1975) and all scored in the normal range for their age (27–30). See Table 1 for details. All participants were compensated for their participation in the study and provided informed consent in accordance with the University of California, Irvine Internal Review Board.

**TABLE 1:** Age (years), education (years), and MMSE (Mini Mental State Exam) for the Intervention group and No-contact control group demonstrates no differences between groups.

	Intervention group	No-contact control group	*t*-value	*p*-value
Age	69.9	71.5	*t*_(__45__)_ = 0.86	*p* = 0.40
Education	14.9	15.9	*t*_(__43__)_ = 1.33	*p* = 0.19
MMSE	28.8	29.0	*t*_(__44__)_ = 0.73	*p* = 0.47

### Experimental Procedure

#### Mnemonic Similarity Task

**FIGURE 1:**
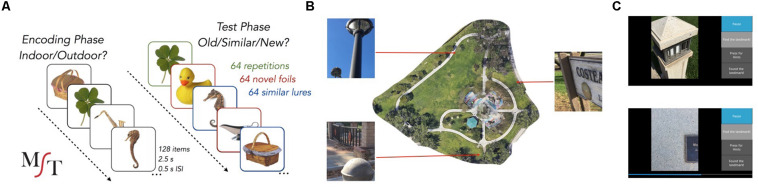
Behavioral task paradigms. **(A)** Schematic of the Mnemonic Similarity Task. **(B)** One of the parks used for the exploration task with three example landmarks and their locations within the park. **(C)** Screenshots from the real-world exploration app.

As part of the behavioral measures, participants completed the MST (Stark et al., 2013) (Figure 1A) at three separate time points (pre-intervention, post-intervention, and follow-up). During the incidental encoding phase, 128 everyday items were presented on the screen, one at a time, for a total of 2.5 s each (0.5 s ISI). Participants made judgments of whether each item was an “indoor” or “outdoor” item by tapping the appropriate response button on a touchscreen laptop. Immediately following encoding, participants performed a recognition memory task for 192 items (64 repeated items, 64 lure items, and 64 foil items) presented for 2.5 s each (0.5 s ISI). Of the 192 items, 64 were repeats from the encoding phase (targets), 64 were similar, but not identical, to an image seen during encoding (lures), and 64 were new (foils). Trial types were presented randomly. Participants again made a response on a touchscreen laptop to judge whether an item was “Old”, “Similar”, or “New”. A LDI was calculated as the difference between the rate of “Similar” responses given to lure items minus the rate of “Similar” responses given to foils. A traditional recognition memory measure was calculated as the difference between the rate of “Old” responses to repeats minus “Old” responses to foils. Participants completed the MST immediately before and after the intervention and again approximately 4 weeks after completing the intervention (average number of days post-intervention 31 ± 6). Thus, we evaluated changes pre- and post-intervention and also whether any observed changes in performance were still present 4 weeks after the completion of the exploration intervention. No-Contact control participants followed the same MST procedure with two sessions administered 4 weeks apart. Importantly, different sets of images (for all conditions) were presented at each timepoint within-subject to reduce potential interference from repeated testing.

#### Real-World Exploration Task

Participants completed a scavenger hunt task in four small community parks (average of 3.3 ± 0.41 acres) in either Laguna Hills, CA or Irvine, CA, United States. The order of the parks was randomized across participants, although an individual would be assigned to either Laguna Hills parks or Irvine parks depending on the proximity to their homes. No participants had prior familiarity with any of the parks used in the study. Participants were provided with a cell phone loaded with the scavenger hunt task application developed by our lab. The task required participants to complete scavenger hunts in four different parks, one park per week, 5 days per park (20 sessions total). There were 16 landmarks in each park (benches, statues, signs, etc.) and each day eight of these landmarks were randomly selected (Figure 1B). On each trial, a cue to the landmark was presented in the form of highly zoomed-in pictures of a component of the landmark (so-as to hide any large contextual cues) and participants were instructed to find and walk to the landmark as quickly as possible (Figure 1C). The app logged GPS coordinates once every second so that we could reconstruct the paths that were taken to each landmark. All participants were trained by the experimenter in how to use the phone and the app on the first day of the intervention. Participants completed the 20 sessions on their own schedule but were only allowed to do one session per day. The app included a hint function and participants were encouraged to use it if they were lost. To do so, they could tap a button and a bar appeared at the bottom of the screen, which filled in from left to right, indicating how close or far away they were from the landmark (Figure 1C bottom). They were told to use this feature if they had already adequately searched for and yet could not find the landmark for that trial.

## Results

### Excess Path

**FIGURE 2:**
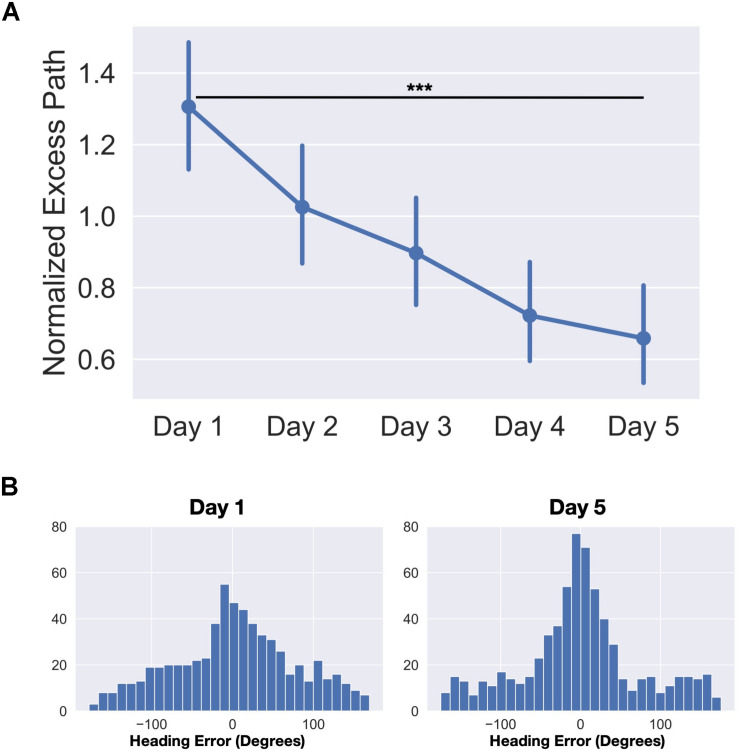
Behavioral data from the real-world exploration task. **(A)** Average normalized excess path (±SEM) across all five days. **(B)** Heading angle errors for day one and day five across the four parks (frequency by trial). ***p < 0.001.

Statistics were performed using a combination of JASP, Prism eight, and Python three. To estimate the amount and rate of learning that occurred over the time in the park, we calculated normalized excess path and heading error for each trial. Trials in which the GPS signal was lost, the app crashed, or there was clear user error (e.g., accidentally closing the app before trial completed) were removed (average 11 trials out of 160 per participant). We then calculated the normalized excess path for each trial by (total path length – ideal path length)/ideal path length. The normalization allows us to compare the excess path of trials where the start of the trial was near the target to those where the target was far away from the start point. We averaged these excess path values across days and parks to control for variability in path due to the random selection of landmarks on each day. We entered average excess path over each of the 5 days into a repeated-measures one-way ANOVA and found a significant main effect of day (*F*_(__4_,_96__)_ = 11.60, *p* < 0.001) (Figure 2A). A planned contrast revealed a significant decrease in excess path from Day 1 to Day 5 (*t*_(__24__)_ = 4.90, *p* < 0.001, *d* = 0.98). The average trial time decreased from an average of 188.1 s on day 1 to 69.9 s on day 5 (averaged across all parks). No differences were observed across parks as the landmarks were similarly distributed and the parks were roughly the same size. Together, these results indicate that participants had learned the locations of the landmarks and that the routes taken to the landmarks became more efficient over time.

### Heading Error

To estimate of how well participants knew which direction to go immediately upon seeing the landmark for that trial, we computed errors in heading angle as the angular difference between the vectors from the start of the trial to the target and from the start of the trial to their position 10 s after the trial began. To determine if the distribution of errors was different from day 1 to day 5, we entered the distributions into a two-sample Kolmogorov-Smirnov test. We found that the distribution of heading error was significantly different on day 5 than it was on day 1 (*D* = 0.11, *p* < 0.001) (Figure 2B). Additionally, we modeled both day 1 and day 5 heading errors as mixture models of a von Mises distribution (circular version of a Gaussian distribution to model angular error in memory for the location) and a uniform distribution (to model random guesses). We computed the spread parameter (*k*) of the von Mises (akin to 1/σ of angular error) for both day 1 and day 5 heading errors which estimates how tightly the distribution is clustered around the mean. We compared the fits of two models, one which constrained the *k* parameters to be equal (*k*_day__1_ = *k*_day__5_) and one where the *k* parameters were allowed to differ between the two models (*k*_day__1_ ≠ *k*_day__5_). We found that the model in which the *k* parameters were allowed to differ significantly increased the quality of fit (*k*_day__1_ = 1.98, *k*_day__5_ = 5.95, difference in AIC = 10.45). These results again highlight the fact that participants learned the locations of the landmarks and by day 5 were heading in the right direction within the first 10 s of the trial.

### Number of Hints

We logged the number of hints participants requested each day. For one participant, the number of hints per day was not accurately recorded, so they were removed from the analysis. We entered the number of hints requested across each of the 5 days in the park into a repeated-measures one-way ANOVA and found a significant main effect of day (*F*_(__4_,_369__)_ = 8.60, *p* < 0.01). Importantly, *post hoc* comparisons revealed a significant decrease in the number of hints from day 1 to day 5 (*t*_(__24__)_ = 3.62, *p* < 0.01, *d* = 0.62), again indicating that participants had learned the landmark locations and required fewer hints to find them by day 5. Overall, the results from the park show that participants had learned the locations of the landmarks in each park and the spatial relationship between them.

### Mnemonic Similarity Task

Our primary endpoint for this intervention was the potential improvement in MST LDI score resulting from the scavenger hunt task. Participants completed the MST both before and after the real-world exploration intervention, as well as at a follow-up appointment approximately four weeks after ending the intervention, with different sets of items used each time. We also tested a no-contact control group that completed the MST twice, approximately 4 weeks apart. We predicted that lure discrimination (LDI) would improve following the intervention, while recognition (REC) memory scores would not change owing to the differential degree of hippocampal involvement in the two measures. We also predicted that our no-contact control group would show no significant improvement on LDI or recognition scores. Four Intervention participants and 1 control participant who performed significantly below chance on the MST’s recognition memory component were removed from the analysis. Here, we removed the entire participant rather than just single timepoint for two reasons: (1) the repeated-measures nature of the design required each timepoint to be present, and (2) we were interested in the effects of the Intervention in healthy, older adults, not those that may be experiencing severe memory deficits. We have employed these criteria in other publications (Stark and Stark, 2017), resulting in a comparable number of removed participants.

**FIGURE 3:**
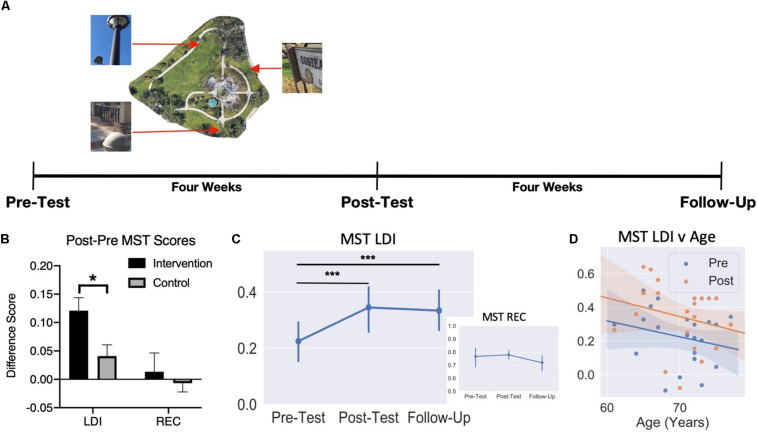
Schematic of behavioral testing timeline. **(A)** Timeline used for behavioral testing. MST was administered at pre-test, post-test, and follow-up for the Intervention Group. **(B)** Improvement in LDI for the Intervention Group compared to Controls, but no difference in REC. **(C)** Average recognition and LDI scores (±SEM) across the three testing sessions. **(D)** Scatter plot of pre-test and post-test MST LDI scores by age. *p < 0.05; ***p < 0.001.

First, we evaluated the primary hypothesis that the Intervention would improve LDI compared to the no-contact controls. An unpaired *t*-test on the difference scores between Pre and Post LDI for each group revealed a greater benefit for the Intervention Group (mean = 0.12) than the No-Contact Control Group (mean = 0.04) (*t*_(__41__)_ = 2.66, *p* < 0.05). We did not find any difference between groups for the difference in REC scores (*t*_(__41__)_ = 0.56, *p* = 0.58) (Figure 3B). Note, follow-up t-tests found that while the pre-post difference score in the Intervention Group (one-sample *t*_(__20__)_ = 5.31, *p* < 0.001) and the Control group were reliably above 0 (one-sample *t*_(__21__)_ = 2.06, *p* = 0.05), the difference between them was a medium effect size (Intervention vs Control Cohen’s *d* = 0.80). Thus, consistent with our observations following video games enrichment, a real-world Intervention succeeded in improving lure discrimination performance on the MST.

To evaluate the trajectory of this effect following a washout period, we ran separate mixed model ANOVAs for recognition and LDI scores with Time as a within-subjects factor (Pre-, Post, and Follow-Up) and Age as a between-subjects factor. For recognition, we observed no significant effect of Time(*F*_(__2_,_16__)_ = 0.99, *p* = 0.39), no effect of Age (*F*_(__12_,_8__)_ = 1.31, *p* = 0.36), and no Age × Time interaction (*F*_(__24_,_16__)_ = 0.67, *p* = 0.82) (Figure 3C). For LDI, we did find a significant main effect of Time (*F*_(__2_,_16__)_ = 12.34, *p* < 0.001) (Figure 3C). Bonferroni-corrected comparisons revealed a significant difference in LDI from pre-test to post-test (*t*_(__20__)_ = 5.24, *p_*bonf*_* < 0.001, Cohen’s *d* = 1.14) as well as a significant difference pre-test to follow-up (*t*_(__20__)_ = 3.98 *p_*bonf*_* = 0.001, *d* = 0.87). There was no significant main effect of Age (*F*_(__12_,_8__)_ = 1.49, *p* = 0.29) or an Age × Time interaction (*F*_(__24_,_16__)_ = 1.78, *p* = 0.12) (Figure 3D). These results indicate that, as we predicted, no change was observed in recognition memory, while LDI improved following the Intervention and remained significantly higher than baseline approximately four weeks after finishing the intervention. The LDI improvement was also observed independent of age, indicating that, within the restricted range of this older sample, being younger does not confer a greater benefit from this training.

### MST and Park Performance

We next wanted to determine how performance in the parks was related to change in LDI. First, for each participant, we computed the slope of their excess path over the 5 days in the park as a metric for how quickly they learned the landmark locations. Overall learning slopes did not differ across the four parks, so we averaged them and then correlated the slope with their change in LDI (post-pre). We found no significant relationship between excess path slope and LDI change (*r*_(__20__)_ = 0.21, *p* = 0.45), indicating that improvement in lure discrimination was possible across many levels of exploration performance. We also did not find any evidence for a significant relationship between the average distance walked per day and change in LDI (*r*_(__20__)_ = −0.116, *p* = 0.62), suggesting that our observed improvement in lure discrimination cannot be explained solely by the amount of physical activity. Finally, we wanted to test whether those who were performing better pre-Intervention had the greatest benefit from the Intervention by correlating pre-Intervention LDI with LDI change (post-pre). We, again, found no relationship between change in LDI score and pre-Intervention baseline (*r*_(__20__)_ = 0.19, *p* = 0.37). Collectively, these results highlight that benefits can be seen from our Intervention regardless of performance level at baseline, how quickly one learns the target locations, or the amount of physical activity engaged in during the intervention. These are important aspects to consider when determining the efficacy of interventions for the larger population.

## Discussion

Here, we sought to determine whether an Intervention based on “environmental enrichment” might improve hippocampal memory ability in humans the way it has in rodents. While humans already lead enriched lives by animal model standards, we have previously demonstrated how exploration of complex 3D video games can improve hippocampal-based memory in both young adults (Clemenson and Stark, 2015; Clemenson et al., 2019) and older adults (Clemenson et al., 2020). Here, we tested whether a real-world exploration intervention could have a similar effect in healthy older adults. Participants completed 20 sessions of a scavenger hunt in four local parks over 4 weeks that required them to learn arbitrary locations within local parks. Excess path measures decreased over time in the parks, as did initial heading error and the number of hints requested, indicating that participants were learning the locations of the landmarks in the park and were able to take more direct paths to them. In addition to simply learning about the parks, the intervention had an effect on their memory ability using a test that is wholly independent of the intervention. Participants showed significant improvement in hippocampal-based mnemonic discrimination (pre-post difference in the MST’s pattern separation based LDI metric), but no significant change in recognition, supporting our hypotheses that environmental enrichment can improve memory ability in older adults. The increase in LDI was not observed in a No-Contact Control Group, again indicating the efficacy of this intervention. Additionally, the boost in LDI was still seen at a 4-week follow-up test for the Intervention Group.

These results are consistent with our previous work in both young (Clemenson and Stark, 2015; Clemenson et al., 2019) and older adults (Clemenson et al., 2020) following environmental enrichment using immersive 3D video games. The magnitude of LDI change in the current study (ΔLDI ≈ 0.1, *d* ≈ 0.8) is consistent with previous studies showing LDI change as a result of a behavioral intervention using video games as a proxy for environmental enrichment in both younger (Clemenson and Stark, 2015; Clemenson et al., 2019) and older adults (Clemenson et al., 2020). In rodents, one effective component of enrichment seems to be the active exploration of the environment (Freund et al., 2013). Our results here, along with our prior work in humans using video games (Clemenson and Stark, 2015; Clemenson et al., 2019, 2020), support the notion that spatial exploration is a viable means of enrichment in humans.

An important difference between the previous work using video games and the current study is that our participants were physically moving in and exploring the environment. Physical exercise has been linked to changes in hippocampal volume and improvements in memory performance in humans (Erickson et al., 2011) and we know that exercise increases neurogenesis and hippocampal memory ability in rodents (Creer et al., 2010), in addition to bolstering surrounding structures such as entorhinal cortex (Vivar et al., 2016). Therefore, it is important to address the role of exercise in a real-world exploration task compared to video game interventions. We saw similar improvements in memory ability as we previously found with a video game intervention, yet we saw no correlation between memory improvement and the average distance walked per day. We cannot conclude that the physical activity inherent in our intervention had no impact. We did, however, find that improvements in memory could be seen independent of the amount of exercise.

Another important finding from the current study is that the observed improvement in memory ability was not dependent on how well one learned the spatial locations during the exploration intervention. This observation is critical for designing interventions for the larger population, since there will be natural variability in how well participants can perform an exploration task. If the intervention only benefits those who are able to learn quickly or start at a higher level of baseline performance, then the intervention is not as useful for the general population. We should note, however, that it is almost certain that participants were incidentally encoding many aspects of the parks as part of their experience during the intervention. We do not know whether variance in how much was learned in these aspects would correlate with the observed improvements in memory ability. Here, we considered the navigation required for this task to constitute free exploration, in contrast to more directed navigation associated with wayfinding (Allison et al., 2016; Voss et al., 2019), but it is possible that we would observe a relationship between navigation performance and improvement in LDI with a more challenging intervention task that required more complex navigation strategies. We also note that memory improvement was observed across all ages in our sample. The age range of our sample is limited, and it is possible that if including a larger age range, we may observe a relationship between participant age and the memory improvement. Given the current results, however, it appears that our intervention can have equal benefit for older adults at any age. Thus, these findings suggest that these benefits can be observed across a range of ability levels, which is critical when designing interventions for wide use in the general population.

Finally, though our intervention required a specific application to complete the scavenger hunt, our methods can be adapted to daily life quite easily. Often, older adults have highly routine schedules and habits that maybe require less spatial learning than would younger adults, who have more flexible and less routine day-to-day experiences. In so far as these results and our prior work support the general notion that enrichment improves hippocampal based memory performance, they suggest alternate avenues for inducing this enrichment. For example, learning the locations of the landmarks in the park and how to navigate between them could translate into taking new routes to the grocery store or a friend’s house. Learning new spatial relationships in this way could mimic the cognitive engagement required for this intervention and on a long-term basis could have significant benefits for hippocampal-dependent memory.

There are certainly several limitations to this study. First, as noted earlier, the role of physical activity should be addressed in future interventions of this type as aerobic exercise has been shown to improve LDI performance (Déry et al., 2013; Heisz et al., 2017). While we found no effect of the amount of distance traveled and while the intervention was not designed to be physically stressful, the effect of walking will warrant future, more detailed research that monitors or manipulates physical activity. Second, we should note that our control group was a passive control. However, in all three of our prior interventions using video games, active controls were used (Clemenson et al., 2015, 2019, 2020) and the no effects were observed there either. Further, a recent meta-analysis has pointed to markedly similar results using passive and active controls in cognitive interventions (Au et al., 2020). Third, future studies should also take care to balance the sample in terms of the number of males and females to be able to account for any sex differences that may contribute to the effectiveness of behavioral interventions.

In any intervention design, we need to be mindful of practice effects on the cognitive test of interest. While test-retest effects can be an issue with many memory paradigms, there are multiple reasons to believe that the benefits we have observed here are not due to repeated testing. First, we have included a no-contact control group, which did not demonstrate improvement following repeated testing using a novel set of items in the MST. Second, our prior intervention work using the MST has also tested control groups in either pre-post (Clemenson et al., 2019) or pre-post-washout (Clemenson and Stark, 2015; Clemenson et al., 2020) designs and, in each of these, there was no reliable effect of time on the LDI measure in controls. Finally, even when we have given explicit instructions prior to the “incidental” encoding task that participants will be tested with an “old/similar/new” paradigm that includes very challenging lure items, there we have observed no change in LDI performance from the surprise recognition version of the task (Stark et al., 2015). The LDI has proven to be a very sensitive measure of hippocampal integrity, while the recognition memory score remains remarkably constant, making it a useful tool in clinical research (Stark et al., 2019). While future intervention-style studies should include other measures of memory and potentially other domains of cognition, test-retest effects should be considered and mitigated to assess improvements based on the intervention.

## Conclusion

We have shown that participating in a 4-week real-world exploration intervention boosted performance on mnemonic pattern separation in older adults, with as little as 10 h of intervention training (approximately 30 min per day, 5 times per week for 4 weeks). These results show a remarkably similar pattern and magnitude of change on MST recognition and LDI scores as seen in our previous work that used video games to provide the spatial exploration and enrichment in younger participants (Clemenson and Stark, 2015; Clemenson et al., 2019) and in older participants (Clemenson et al., 2020). Importantly, our intervention can be easily implemented into a daily schedule and potentially alleviate some of the age-related decline in hippocampal-dependent memory.

## Data Availability Statement

The datasets generated for this study are available on request to the corresponding author. The data will be made available upon request from the corresponding author.

## Author Contributions

BK, SS, and CS contributed to the design of the study. BK created the app and real-world task, collected and analyzed the data. BK, SS, and CS contributed to the interpretation of the results and writing the manuscript.

## Conflict of Interest

The authors declare that the research was conducted in the absence of any commercial or financial relationships that could be construed as a potential conflict of interest.
